# A Facile and Promising Delivery Platform for siRNA to Solid Tumors

**DOI:** 10.3390/molecules29235541

**Published:** 2024-11-23

**Authors:** Qixin Leng, Aishwarya Anand, A. James Mixson

**Affiliations:** Department of Pathology, University of Maryland School of Medicine, 10 S. Pine St., Baltimore, MD 21201, USA; qleng@som.umaryland.edu (Q.L.); aishwaryaanand@som.umaryland.edu (A.A.)

**Keywords:** siRNA, polyplex, nanoparticle, histidine, lysine, peptide, cancer

## Abstract

Over 20 years have passed since siRNA was brought to the public’s attention. Silencing genes with siRNA has been used for various purposes, from creating pest-resistant plants to treating human diseases. In the last six years, several siRNA therapies have been approved by the FDA, which solely target disease-inducing proteins in the liver. The extrahepatic utility of systemically delivered siRNA has been primarily limited to preclinical studies. While siRNA targeting the liver comprises relatively simple ligand-siRNA conjugates, siRNA treating extrahepatic diseases such as cancer often requires complex carriers. The complexity of these extrahepatic carriers of siRNA reduces the likelihood of their widespread clinical use. In the current report, we initially demonstrated that a linear histidine–lysine (HK) carrier of siRNA, injected intravenously, effectively silenced luciferase expressed by MDA-MB-435 tumors in a mouse model. This non-pegylated linear peptide carrier was easily synthesized compared to the complex cRGD-conjugated pegylated branched peptides our group used previously. Notably, the tumor-targeting component, KHHK, was embedded within the peptide, eliminating the need to conjugate the ligand to the carrier. Moreover, brief bath sonication significantly improved the in vitro and in vivo silencing of these HK siRNA polyplexes. Several other linear peptides containing the -KHHK- sequence were then screened with some carriers of siRNA, silencing 80% of the tumor luciferase marker. Additionally, silencing by these HK siRNA polyplexes was confirmed in a second tumor model. Not only was luciferase activity reduced, but these siRNA polyplexes also reduced the Raf-1 oncogene in the MDA-MB-231 xenografts. These simple-to-synthesize, effective, linear HK peptides are promising siRNA carriers for clinical use.

## 1. Introduction

Similarly to the current excitement surrounding Crispr/Cas9 therapies, small interfering RNA (siRNA) aroused comparable sentiments at the beginning of the millennium. In 2002, the Science magazine selected siRNA as the “Molecule of the Year” [1], and several years later, the Nobel Prize in Medicine was awarded to Andrew Fire and Craig Mello [2]. siRNA can silence cellular mRNA genes in diverse eukaryotes in the plant and animal kingdoms [3]. Consequently, siRNA technology and therapeutics have been used to modify gene expression, from enhancing the nutritional value of plants [4] to treating human diseases [5].

Thus far, FDA-approved systemic siRNA therapies have exclusively targeted the liver for two reasons. First, several mRNAs and their proteins expressed in the liver cause or enhance the predisposition to develop a disease. By reducing the level of these mRNAs, siRNA therapy can effectively minimize various disease symptoms and signs. These diseases include acute hepatic porphyria, transthyretin amyloidosis, and heterozygous familial hypercholesteremia (hFH) [5,6,7]. With coronary heart disease, a serious health problem in America, lowering LDL cholesterol levels in patients who do not respond to statins has received significant attention. By reducing PCSK9 levels in hepatocytes, siRNA therapeutics can lower persistently elevated LDL cholesterol in statin-receiving patients with hFH or atherosclerotic heart disease [8,9]. In contrast to bi-monthly antibodies targeting PCSK9, siRNA maintenance therapy can be administered every six months [10].

Second, and perhaps more importantly for targeting the liver, there are efficient delivery systems for siRNA to the hepatocyte. When conjugated to siRNA, a trimer N-acetyl galactose ligand, GalNAc, results in the prolonged silencing of the target mRNA and protein in the liver. The GalNAc-siRNA conjugate is endocytosed into the liver hepatocytes by binding with high specificity to the cell surface asialoglycoprotein receptors [11]. Furthermore, these injectable GalNAc-siRNA conjugates are simple in their design, which can facilitate their upscale production for general clinical use. Because of their increased efficacy and simplicity in design, there has been a shift from the liposomal delivery of siRNA to the GalNAc conjugates in recent years [12]. The success of siRNA targeting various liver mRNA underscores this treatment strategy.

In contrast to the liver, there are no FDA-approved extrahepatic therapies for systemically delivered siRNA, including tumors [13]. In preclinical studies, numerous tumor targets, such as oncogenes and checkpoint inhibitors, have been and could be targeted and silenced by siRNA therapy [14]. These targets are in the tumor cells and their supporting cells, such as endothelial cells and immune cells. By silencing tumor-promoting genes, siRNA therapy can regress the size of tumors. Although there is no paucity of targets, the biggest obstacle to using siRNA for treating human cancer is the lack of an effective systemic carrier system. There are several factors, such as instability of the nanoparticle, short half-life in the bloodstream, and poor entry of nanoparticles into the tumor. Most nanoparticles, including polyplexes, depend on the EPR effect, which frequently results in the unreliable and inconsistent delivery of the nanoparticle with few successes in clinical trials [15,16,17]. Considerable efforts for extrahepatic diseases have consummated few successes, all of which have incorporated chemotherapy agents and not nucleic acids, including siRNA [17,18,19].

Another problem of delivering siRNA to tumors is the high degree of complexity of the nanoparticles. Much like a layered cake, many carrier-siRNA nanoparticles have layer after layer of complexity to release the drug or siRNA into the tumor environment. These nanoparticles often incorporate a drug and siRNA in preclinical studies [17,20]. Due to the complexity of synthesis and formulations, these carrier systems often lack reproducibility, and upscaling these carrier systems to large clinical trials is expensive and may be a limiting factor to their widespread use in human cancer. As a result, most carriers that carry siRNA effectively to tumors in animal models may not achieve prevalent usage in humans.

Despite many laboratories, including ours, focused on developing systemic nucleic acid delivery systems for several decades, progress has been slow. What is needed is a non-viral carrier that is simple to synthesize and non-toxic, which effectively delivers the therapeutic nucleic acid systemically to tumors. We have recently discovered a series of linear peptides that fulfilled those criteria. These linear histidine–lysine (HK) peptides were effective carriers of siRNA, primarily because the tumor-targeting component was embedded within the peptide. In contrast to most nanoparticles, including polyplexes, the HK siRNA polyplex does not require the enhanced permeability and retention (EPR) effect to enter the tumor. By targeting the neuropilin-1 transport system, the peptide component of the siRNA nanoparticle enabled extensive distribution of the therapeutic siRNA throughout the tumor. Our long-term goal is to develop a siRNA delivery platform targeting oncogenes and checkpoint inhibitors as a cancer therapeutic.

## 2. Results

### 2.1. In Vitro Transfection

**Figure 1:**
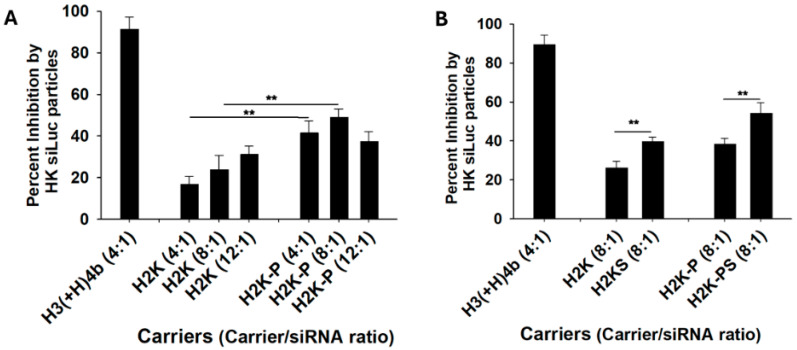
In vitro silencing with HK siLuc polyplexes. (**A**) After MDA-MB-435-Luc cells, increasing peptide amounts (ranging from 4 to 12 μg) were mixed with siLuc (1.0 μg) in Opti-MEM and formed for 45 min. The polyplexes were then added to the well. After 48 h, the luciferase activity was measured. **, *p* < 0.01, H2K-P > H2K (4:1 and 8:1 ratios); Student’s *t*-test. The 4:1 *w*/*w* ratio of H3K(+H)4b, H2K, and H2K-P siRNA polyplexes corresponds to an N/P ratio of 5.4, 6.1, and 5.5, respectively. (**B**) Sonication effects. After H2K or H2K-P was mixed with the siRNA (8:1 ratio), half the samples were sonicated for 1 min (H2KS and H2K-PS). The polyplexes, formed for 45 min, were added to the cells, and luciferase levels were measured 48 h later. **, *p* < 0.01, H2KS > H2K; H2K-PS > H2K-P; Student’s *t*-test. The un-sonicated H3K(+H)4b siLuc polyplexes were the control.

We determined that the polymerized H2K-P as a carrier of siLuc improved luciferase silencing compared to the H2K carrier (Table 1, Figure 1A) The silencing differences between these two carriers were particularly marked with the 4:1 and 8:1 ratios of the HK siRNA polyplex. The H2K-P at the 4:1 ratio silenced luciferase activity by 42%, while the H2K peptide silenced luciferase by 16%. Still, the linear carriers, H2K and H2K-P, were significantly less effective than the branched carrier, H3K(+H)4b. The 4:1 ratio of H3K(+H)4b:siRNA was selected based on prior in vitro results demonstrating 90% or greater silencing of the luciferase marker [21]. The three HK polyplexes prepared in Opti-MEM were retained in the well during gel electrophoresis (Figure S1). Because we previously found that brief sonication of lipo-HK-peptides of siRNA improved silencing (unreported findings), we investigated whether sonication improved silencing with linear H2K carriers (Figure 1B). Both sonicated H2K- and H2K-PS siLuc polyplexes markedly reduced luciferase activity compared to un-sonicated polyplexes.

### 2.2. The Polymerized Carrier, H2K-P, of siRNA Efficiently Silenced a Tumor Marker In Vivo

**Figure 2:**
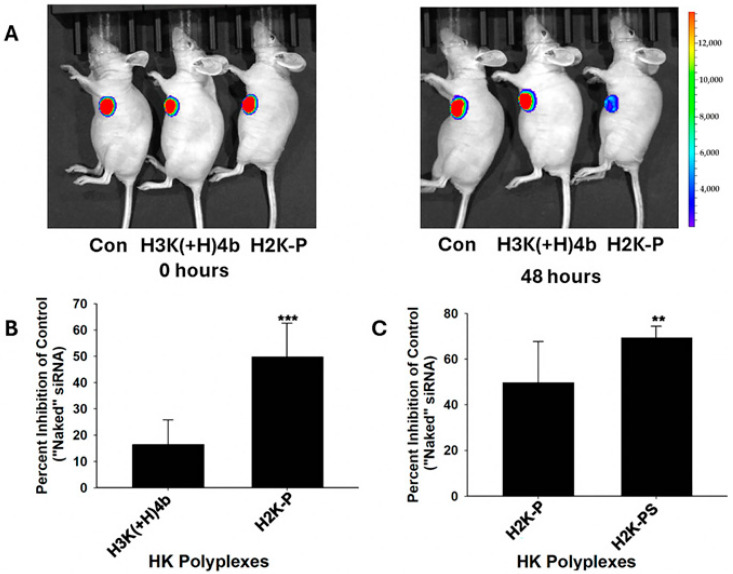
H2K-P markedly decreased luciferase activity in vivo. (**A**) Luciferase activity of tumors was determined at 0 h and 48 h after injection of H2K and H2K-P siLuc polyplexes. (**B**) Inhibition of luciferase activity by H3K(+H)4b or H2K-P polyplexes. ***, *p* < 0.001; Student’s *t*-test (*n* = 4). (**C**) Bath sonication improved the silencing activity of H2K-P siLuc NP. H2K-PS, sonicated H2K-PS vs. un-sonicated H2K-P polyplexes. **, *p* < 0.01; Student’s *t*-test (*n* = 8).

We then investigated the effectiveness of the H2K-P carrier of siRNA in a mouse model in which tumors were grown to between 100 and 150 mm^3^ (Figure 2). In this study, a melanoma tumor expressed the luciferase marker (MDA-MB-435-Luc), and our objective with the siRNA carrier was to decrease the luciferase marker. A red-to-blue color shift on the visual light spectrum scale determined greater silencing of the tumor’s luciferase at 48 h. We initially compared the in vivo silencing efficacy of systemically delivered H3K(+H)4b and H2K-P siLuc polyplexes (Figure 2). While H3K(+H)4b siLuc polyplexes reduced luciferase activity by about 14% in MDA-MB-435-Luc tumors, H2K-P polyplexes reduced activity by nearly 50% after one injection (Figure 2A,B).

Notably, there was no correlation between the in vitro and in vivo efficacy of H3K(+H)4b and H2K-P polyplexes. The H3K(+H)4b siLuc polyplex, which silenced the luciferase marker in vitro by 90%, minimally reduced the tumor’s luciferase activity in vivo (Figure 1, Table 2). In contrast to the H3K(+H)4b and H2K-P polyplexes prepared in water (Figure S2A), the H2K polyplex was not retained during electrophoresis in the well with HK/siRNA ratios of 1.5:1 to 6:1 (*w*:*w*) (Figure S2). Not surprisingly, the H2K carrier did not effectively silence luciferase expressed by the MDA-MB-435 xenograft (Table 2).

### 2.3. Brief Sonication Significantly Improved Silencing Activity of siRNA Polyplex In Vivo

Based on the in vitro studies (Figure 1B), we examined whether sonication improved the silencing of luciferase in tumors in vivo (Figure 2C, Table 2). The sonicated polymerized H2K-PS polyplex silenced tumor luciferase expression markedly compared to the un-sonicated H2K-P. The size and PDI of the sonicated and un-sonicated polyplexes were not markedly different (Table S1). Because of the improvement in silencing, the remainder of the HK polyplexes tested for silencing efficacy were sonicated before injection in vivo.

### 2.4. NRP-1 Dependent H2K-P siLuc Polyplexes

**Figure 3:**
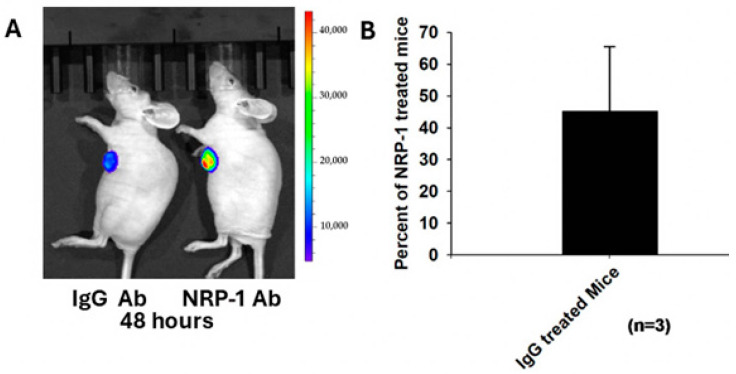
NRP-1 transport mediates silencing by H2K-P polyplexes. Tumor-bearing mice were pre-injected with IgG or NRP-1 antibodies 60 min before injecting the HK polyplexes. Forty-eight hours later, the mice were imaged, and the luciferase activity of the tumors was measured by the imaging software. (**A**) Representative image of the MDA-MB-435 tumor-bearing mice pre-treated with the antibodies before the polyplex injection. (**B**) IgG-treated mice had a 54.8% reduction in luciferase activity compared to NRP-1-treated mice.

In contrast to the H3K(+H)4b peptides with a repeating sequence of -K-H-H-H-K-, H2K-P has a repeating sequence of -K-H-H-K (Table 1). Because the NRP-1 receptor, up-regulated in tumor endothelial and tumor cells (including MDA-MB-435 and MDA-MB-231), binds to the -K-X-X-K- sequence [23,24], we investigated whether this receptor transport system had an essential role in the tumor uptake of H2K-P siRNA polyplexes. Pre-injecting an antibody that blocks the NRP-1 receptor before the H2K-P siLuc polyplex markedly reduced the silencing activity in vivo (Figure 3). In contrast, the silencing activity of the H2K-P polyplex was maintained by pre-injecting with the controlled IgG antibody and had 45% of the luciferase activity of the NRP-1 pre-treated mice. Consequently, because of the importance of the NRP-1- directed sequence, the H3K(+H)4b peptide with a predominant sequence of -K-H-H-H-K- was surprisingly not an ineffective carrier of siLuc in vivo (Figure 1, Table 2).

### 2.5. Screening and Discovering Increasingly Effective Carriers of siRNA

**Figure 4:**
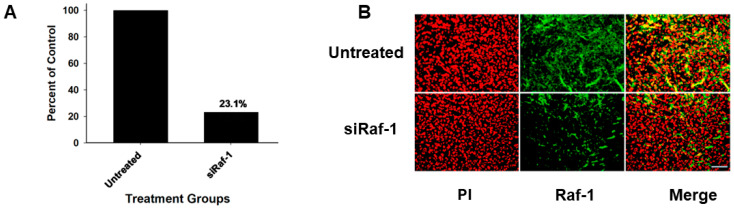
Raf-1 levels markedly reduced. (**A**) mRNA levels. Forty-eight hours after injection of siRaf-1 (40 μg) in complex with the H3K-33/H2K-CO_2_H (60 μg, 15% H2K-CO_2_H), mRNA levels were isolated from treated and untreated MDA-MB-231 tumor-bearing mice, Raf-1 mRNA levels in the siRaf-1 polyplex-treated mice were 23.1% of untreated mice by quantitative PCR (*n* = 2). (**B**) Raf-1 oncoprotein levels. Similarly, Raf-1 levels were significantly decreased in the siRaf-1 polyplex-treated group. PI, propidium iodide. Bar, 100 μm.

As a result of the NRP-1 transport system playing a major role in the transport of these polyplexes, other HK peptides with a sequence of -KHHK- were tested for their ability to silence the tumor luciferase marker in vivo. Most linear and branched HK with -KHHK- carriers of siLuc silenced the tumor marker effectively. In particular, the H3K-33/H2K-CO_2_H combination carrier showed the most efficacy in reducing luciferase activity in MDA-MB-435 and MDA-MB-231 tumor xenografts (Table 2, Figure S3). In addition to luciferase, the combination (H3K-33 (85%)/H2K-CO_2_H) markedly reduced endogenous Raf-1 mRNA (23.1% of control, 76.9% inhibition) and oncoprotein similarly in an MDA-MB-231 tumor-mouse model (Figure 4).

The poorest HK carriers for silencing with the -KHHK- sequences were the linear H2K and the H3K-33 peptides. As stated, the linear H2K carrier (and the related H2K-CO_2_H) did not form stable siRNA polyplexes when prepared in water (Figure S2). It is not evident why the H3K-33 carrier was ineffective since it contained K-H-H-K sequences (Table 1) and formed stable polyplexes (Figure S2). The maximal amount of H2K-CO_2_H added to the H3K-33 carrier is probably 25% since a minor quantity of siRNA was released from the polyplex during electrophoresis (Figure S2). The size varied from 107 nm to 325 nm, with the largest HK polyplexes having the H3K-33 component (Table S1).

## 3. Discussion

Non-viral carriers have been developed to carry different nucleic acids such as mRNA, plasmids, and siRNA. These various nucleic acids require different non-viral carriers and nanoparticle designs [25,26]. The designs of the particles are controlled by balancing the intracellular release of the nucleic acid with the stability of the polyplex and nucleic acid required during their circulatory transit to the tumor [26]. Moreover, in vitro transfection with carriers often does not correlate with in vivo transfection. Although H2K-P was a significantly more efficient carrier of plasmids in vitro, H2K and H2K-P were comparable in transporting plasmids in vivo [22].

In contrast, there were marked differences between these two HK carriers of siRNA for tumor delivery in vivo (Figure 2). The inadequate silencing by the H2K carrier was probably due to the lack of stable siRNA polyplexes, even with the relatively high peptide/siRNA ratio of 6:1 (Figure S2). The H2K-P in complex with siRNA formed stable polyplexes at low ratios as demonstrated by their complete well retention during the gel retardation assay (1.5:1). Since the H2K-P and H2K carriers have similar histidine and lysine patterns and N/P ratios (Table 1 and Table 2), the enhanced stability of the H2K-P polyplex was likely due to the higher molecular weight and greater interactions of the polymerized carrier with the siRNA. Similarly to the H2K-P polyplex, the 33-mer H3K-33 in complex with siRNA was completely retained in the well of the gel during electrophoresis, suggesting a critical length of the peptide for stabilization (Figure S2) [22,27].

We previously developed a targeted HK siRNA polyplex that reduced the tumor luciferase by 70% [21,28]. Biodistribution studies showed the enhanced tumor accumulation of the targeted polyplex compared to the non-targeted polyplex. Like other nanoparticles and polyplexes [29,30], these targeted siRNA polyplexes showed significant uptake by the liver and spleen of the targeted and untargeted polyplexes. Although the carrier was effective in its ability to carry siRNA to the tumor, the complexity of this carrier may be an obstacle to upscaling the product to clinical trials [28]. The siRNA carrier comprised a cRGD-pegylated four-branched carrier and an unmodified H2K4b four-branched carrier. The four branched carriers were expensive and difficult to synthesize, particularly for the full-length sequence of the terminal branch. The modification of these carriers with PEG and cRGD further complicated the synthesis. Because of the complexity of the carrier, we sought to develop a less expensive carrier that could be readily upscaled for widespread clinical use.

Of the effective linear and branched HK carriers of siRNA (Table 2), the H2K-P carrier was one of the easiest to prepare and was effective in vivo. The H2K-P product, oxidized from the C-H2K-C monomer, represents a mixture of polymers with an average molecular weight of 19,170 Daltons [22]. The lack of a defined product may be problematic or lead to delays with the FDA. Furthermore, the refinement and purification of these concatemers of C-H2K-C led to the significant loss of the original polymerized product. Our early attempts to purify the polymerized product with HPLC reduced its transfection ability of plasmids [22]. These potential problems may be minimized by utilizing filtration units with larger molecular weight exclusions or other purification methods [31].

Because of potential problems with H2K-P, we also investigated several peptide carriers with defined molecular weights. Several of these -KHHK-containing peptides, including linear and branched HK carriers, showed promise as carriers of siRNA (Table 2). Silencing in vivo with the -KHHK- containing carriers of siRNA ranged from 25.7% to 81.6%, excluding the linear H2K carrier, which did not form a stable polyplex. Whereas linear peptides were relatively easy to synthesize, branched polymers such as H2K4b-14 were challenging to synthesize and would likely increase the product’s costs. Interestingly, the H3K-33 peptide with consecutive -KHHK-sequences was an inadequate carrier for siRNA (Table 2). The reason for this is unclear, but perhaps the HK carrier requires more than two consecutive sequences. Of the linear HK carriers, the H3K-33/H2K-CO_2_H combination is an attractive candidate to explore further based on its silencing efficacy and biophysical characteristics. While H3K-33 alone was an ineffective carrier, adding H2K-CO_2_H to the H3K-33 peptide greatly enhanced the polyplexes’ silencing in vivo. The combination carrier, H2K-CO_2_H/H3K-33, demonstrated a marked reduction in the luciferase in two tumors xenografts and the significant inhibition of the Raf-1 oncogene in MDA-MB-231 tumors (Table 2; Figure 4 and Figure S3).

The H2K-CO_2_H component was not added to other carriers, but we suspect its addition to the other carriers (i.e., H2K-P) could enhance their silencing ability in vivo. The NRP-1 receptor, up-regulated in tumors, recognizes R/KXXR/K peptide, where R/K represents either arginine or lysine and X represents any amino acid. In addition, binding to the NRP-1 receptor requires that this peptide sequence at its C-terminal end has a carboxyl group [23,32]. Consequently, H2K-P (and most other -KHHK-containing peptides) requires limited enzymatic digests to expose the C-terminal carboxyl groups for NRP-1-mediated polyplex uptake into the tumor. Although multiple enzymes within the tumor matrix can process HK peptides to bind to the NRP-1 receptor [33,34,35,36], the required processing of the peptides may decrease NRP-1-mediated uptake into the tumor [37]. Consequently, the silencing enhancement achieved by adding H2K-CO_2_H suggests that enzymatic activation can be a limiting factor, at least with the H3K-33 peptide. The NRP-1 transport system can recognize the H2K-CO_2_H component without any enzymatic modification.

Because the dependence of NPs to accumulate in tumors by the EPR effect has clinically proven ineffective with few exceptions [17], alternative pathways such as the NRP-1 transport pathway should be investigated. Although not universally expressed in tumors, NRP-1 is expressed in a sizable portion of solid tumors. For example, breast cancers with high EGF activity have increased NRP-1 [38]. The more aggressive breast cancers, such as triple-negative and Her-2 positive tumors, often have high levels of NRP-1 [38]. In addition to breast cancers, between 40 and 50% of pancreatic tumors have elevated NRP-1 levels [39]. Moreover, based on animal data, the angiogenic tumor endothelium expresses elevated levels of NRP-1 [32,33]. The NRP-1 transport system enables the widespread distribution of small to large molecules through transcytosis or increased vascular permeability [39,40,41,42]. Whereas EPR-based polyplexes optimally require a size of less than 100 nm [43], the NRP-dependent polyplexes in our study were less constrained by size. It remains unclear whether NRP-directed NP will be effective in tumors (i.e., tumor cells) that do not express NRP-1, but the tumor endothelium does. Because the tumor endothelium represents a significant barrier to NP, this study may be interesting and have clinical significance.

One would not think that brief bath sonication would influence the silencing efficacy of non-malleable, relatively small-sized polyplexes. Despite ultrasound not appreciably affecting the size, PDI, or zeta potential of H2K-PS siRNA polyplexes, the sonication of these polyplexes (before addition to cells) increased the silencing efficacy in vitro and in vivo (Figure 1 and Figure 2; Table 3 and Table S1). In contrast to polyplexes, the malleable membranes of liposomes and cells are significantly affected by sonication, particularly in the presence of microbubbles. Consequently, sonication has previously increased the transfection of plasmid DNA, lipoplexes, and polyplexes, primarily by cavitating bubbles that create pores in the cellular membrane [44,45]. Ultrasound has also been used to break up large aggregates of lipoplexes to increase transfection [46], but un-sonicated polyplexes in the current study did not form large aggregates, nor was there evidence of size reduction with brief sonication. Although the exact mechanism is unclear, it does appear that sonication affects the structure or interactions within these siRNA polyplexes to enhance silencing in vitro and in vivo. It would be interesting to determine if sonication enhances the transfection of HK plasmid polyplexes, like the siRNA polyplexes. Further studies are required to understand the mechanisms of sonication for the improved silencing of these siRNA polyplexes.

## 4. Materials and Methods

### 4.1. Animals

Female athymic mice (4–8 weeks old) were purchased from Envigo (Indianapolis, IN, USA). The experiments were approved by the Institutional Animal Care and Use Committee of the University of Maryland Baltimore. The protocol was approved on 27 May 2022 with the protocol number 0322009.

### 4.2. Cell Lines

Two human malignant cell lines, MDA-MB-435 and MDA-MB-231, purchased from ATCC (Manassas, VA, USA), stably expressed firefly luciferase (MDA-MB-435-Luc and MDA-MB-231-Luc) [21]. These cells and MDA-MB-231 cells were cultured in Dulbecco’s minimal essential medium containing 10% fetal calf serum and 20 mM glutamine.

### 4.3. Polymers

Linear and branched HK peptides were synthesized on a Ranin Voyager peptide synthesizer (Tucson, AZ, USA) by the biopolymer core facility at the University of Maryland or by Genscript (Piscataway, NJ, USA), as previously described (Table 1) [47]. Peptides were analyzed by high-performance liquid chromatography (Beckman Coulter, Fullerton, CA, USA) or ESI mass spectroscopy (LCMS-2020, Shimadzu Corporation, Kyoto, Japan) to ensure a purity of 90% or greater. The lyophilized peptides were reconstituted in RNAse/DNase free water (Corning, Manassas, VA, USA) at a concentration of 30 mg/mL, as previously described [47]. The polymerization of C-H2K-C resulting in H2K-P (also named H2KC-48) was performed as previously reported [22]. In brief, C-H2K-C (5 mg) was oxidized by DMSO (30%) in HEPES buffer for 48 h and then capped with S-methyl methane (80 mM) for 30 min. The polymerized H2K-P was further purified by filtering out lower molecular weight polymers with an Amicon 0.5 mL 10 kDa cutoff filter (Millipore Sigma, Darmstadt, Germany). The MW of H2K-P was 19,170 Daltons [22]. The branched HK polymers, H3K(+H)4b and H2K4b-14, have four branches emanating from a 3-lysine core.

### 4.4. siRNA

The sequences of siRNA targeting luciferase (siLuc) were as follows: sense, 5′-CUG-CAC-AAG-GCC-AUG-AAG-A-dTdT-3′; antisense, 5′-UCU-UCA-UGG-CCU-UGU-GCA-G-dTdT-3′ [21,28]. Those targeting Raf-1 (si-Raf-1) were as follows: sense, 5′-UGU-CCA-CAU-GGU-CAG-CAC-C-dTdT-3′; antisense, 5′-GGU-GCU-GAC-CAU-GUG-GAC-A-dTdT-3′ [48].

### 4.5. In Vitro HK siRNA Polyplex Formation for Luciferase Silencing

MDA-MB-435 cells expressing Luc (MDA-MB-435-Luc) were plated in a 24-well plate (0.5 mL of Dulbecco’s minimal essential medium, 10% serum) at a density of 3 × 10^4^ cells per well. Various peptides and peptide combinations (H2K, H2K-P), in complex with Luc-siRNA (1 μg), were prepared in Opti-MEM, a media that enhances cellular delivery of polyplexes (Invitrogen, Thomas Fisher Scientific, Waltham, MA, USA) [21,22,47,49]. After mixing HK with siRNA in Opti-MEM, the polyplexes were formed for 45 min before they were added dropwise to the MDA-MB-435 cells [50]. In some experiments, the polymer and siRNA, after mixing, were sonicated for 1 min (Bath Sonicator, 40 Hz, 60 W, Lashio, Shaoxing, China) before letting the solution stand for 45 min. Based on prior studies to evaluate silencing and transfection, the HK to siRNA ratios for the in vitro experiments were 4:1, 8:1, and 12:1 (*w*:*w*, weight/weight) [22,47,50]. After the polyplexes were incubated with the cells for 48 h, the cells were lysed (200 μL of Luciferase Lysis Cell Buffer, 5×; Promega, Madison, WI, USA) followed by centrifugation at 12,500 RPM for 5 min. The luciferase activity in the supernatant fraction was measured by a Turner TD 20/20 luminometer (Turner Design, Sunnyvale, CA, USA).

### 4.6. In Vivo HK siRNA Polyplex Preparations

For tumor xenograft experiments in vivo, mice were treated by injection in the tail vein with HK polyplexes containing 40 μg of siLuc or siRaf. After the polymers (60 μg) were mixed with the siRNA, the resulting polyplex formed at room temperature for 40 min. In some instances, after mixing HK peptides with siRNA, the polyplex solution was sonicated for 1 min and then left to stand for 40 min before it was injected. Each mouse was injected intravenously with 230 μL of the polyplexes. The size of the polyplex and gel retardation of siRNA (40 μg) by the minimal amount of peptide determined the ratio for in vivo use. The HK to siRNA ratios were expressed as weight to weight (*w*:*w*) or as N/P ratios [26,51]

### 4.7. Gel Retardation Assay

Like the in vivo polyplex preparations, various HK peptides (60 μg) were mixed with siRNA (40 μg) in water (total volume of 230 μL) and incubated for 40 min at room temperature. Some polyplex preparations were sonicated for 1 min before they were left to stand for the time remaining. After the HK siRNA polyplex (10 μL, 1.74 μg) was loaded onto the gel (3% agarose), electrophoresis was carried out at a constant voltage of 50V for 30 min in Tris-Acetate-EDTA (TAE) buffer containing ethidium bromide. The siRNA band densities were then visualized with an ultraviolet system (ChemiDoc Touch, BIO-RAD, Hercules, CA, USA). Part of the polyplex solution (30 μL) was used to measure the size and surface charge. Similarly, HK polyplexes prepared for in vitro use were analyzed with gel electrophoresis.

### 4.8. Particle Size and Surface Charge

The size, PDI, and zeta potential of HK siRNA polyplexes prepared were determined with a Zetasizer (Malvern, Westborough, MA, USA) and analyzed with the instrument manufacturer’s software (Zetasizer software, version 6.2). Using dynamic light scattering at a 90° angle, the particles’ sizes were reported as the Z-average diameter from the intensity-weighted size distribution. After the size and PDI of the HK polyplexes (30 μL of the polyplex solution + 200 μL of water) were measured, the zeta potential was determined after adding 800 μL of 10 mM NaCl solution to the polyplex.

### 4.9. In Vivo Bioluminescence Experiments

MDA-MB-435-Luc or MDA-MB-231-Luc xenografts were established by injecting 4 × 10^6^ cells in the midclavicular line or mammary fat pad of female nude mice (Envigo). After the tumor reached between 100 and 150 mm^3^, mice with equal tumor volumes were usually separated into three treatment groups: siLuc control and two HK siLuc polyplex groups (1.5:1 ratio). These polyplexes containing an HK peptide (60 μg) and siLuc (40 μg) were administered in the tail vein. At 0 h and after 48 h, the mice were anesthetized and injected i.p. with 150 mg/kg of the D-Luciferin substrate (Caliper Life Sciences, Hopkinton, MA, USA). The tumors’ luciferase levels were then measured by the IVIS-200 optical image system (Caliper Life Sciences). The Living Imaging software (Caliper Life Sciences, version 4.7.4) determined the region of interest for photon emission measurement. Tumor volume was determined using the formula ½ × length × width^2^.

### 4.10. Quantitative PCR for Raf-1

At 48 h after the MDA-MB-231 tumor-bearing mice (100 to 150 mm^3^ in size) were injected with the siRaf-1 polyplexes, the tumor was divided for mRNA and immunohistochemical staining analysis. The tumor RNA from the untreated and treated mice was isolated using the RNeasy mini kit according to the manufacturer’s instructions (Qiagen, Germantown, MD, USA, Cat#74104). The concentration and quality of RNA of an untreated and treated (polyplex containing Raf-1 siRNA) tumor were analyzed using a nanodrop and adjusted to obtain similar RNA concentrations. Using this RNA, cDNA was prepared with BioLabs reverse transcription kit (LunaScript^®^ RT SuperMix Kit; New England Biolabs, Ipswich, MA, USA, Cat#E3010G), according to the manufacturer’s instructions. The qPCR mix contained reverse/forward primers (2 μL, 100 μM), 10 μL of 2× Sybr Green master solution (Bimake, Houston, TX, USA), 2 μL of cDNA, and 6 μL of nuclease-free water. The primer sequences for Raf-1 are below (Table 3). qPCR was carried out with the Bio-Rad CFX96 Real-Time System (Bio-Rad, Hercules, CA, USA) with the following PCR conditions: an initial denaturing step of 95 °C for 3 min, then 39 cycles of 95 °C for 10 s and 60 °C for 30 s. Gene expression levels were analyzed using Livak’s formula, and all levels were normalized against the housekeeping GAPDH.

### 4.11. Immunohistochemical Staining for Raf-1 of Tumors

Tumors were fixed in 10% formalin for 24 h and processed as paraffin-embedded tissue sections. Immunostaining was performed as previously described [28]. For detection of Raf-1, tissue sections were incubated with rabbit antihuman polyclonal Raf-1 antibody (Sino Biological US Inc., Wayne, PA, USA, Cat# 101053-T08), and then goat anti-rabbit IgG secondary antibody labeled with FITC (Thermo Fisher Scientific, Waltham, MA, USA, Cat# 65-6111). Images were obtained with the E2000-S microscope (Nikon, Tokyo, Japan).

### 4.12. NRP-1 Dependent Silencing of Luciferase Expression by Tumors

Tumor-bearing mice were administered intravenously with NRP-1 antibody [R&D Systems, Minneapolis, MN, USA; 6 μg in 200 μL of phosphate-buffered saline (PBS)] or an immunoglobulin (Ig)G antibody control (6 μg in 200 μL of PBS) 60 min before the injection of H2K-P siLuc polyplexes (*n* = 3). Forty-eight hours later, the mice were imaged with the IVIS to assess the luciferase expression of the tumors.

### 4.13. Statistics

Results, reported as mean ± standard deviation (±SD), represent three or more separate data measurements for MDA-MB-435 cells and tumors. For MDA-MB-231 tumors, each experiment was repeated (quantitative PCR, immunohistochemical staining, and luciferase suppression) (*n* = 2). Results for the MDA-MB-435 cells and tumors were analyzed using a two-tailed *t*-test. A double asterisk represents *p* < 0.01, and a triple asterisk represents *p* < 0.001 (SigmaPlot, San Jose, CA, USA).

## 5. Conclusions

In summary, a series of HK siRNA polyplexes with an NRP-1 tumor-targeting component reduced luciferase levels in two tumor models. The reduction in the luciferase marker by these HK siRNA polyplexes corresponded to the reduction in the tumor Raf-1 oncogene. Compared to the branched HK peptides, the effective linear HK carriers were easy to synthesize and are promising candidates to expedite the development of siRNA polyplexes for treating human cancer. [21,28]. Moreover, the direct sonication of the H2K-PS siRNA polyplex improved the silencing significantly, although the mechanism is unclear and requires further investigation. In contrast to the EPR-dependent polyplexes, these NRP-1-dependent polyplexes are less dependent on the size for their tumor uptake. The best of these linear HK carriers showed at least equivalent delivery of siRNA compared to the more complicated and modified pegylated peptide carriers.

We envision two related applications for these NRP-1-dependent HK carriers for siRNA. Target validation is one area this siRNA platform may expedite since few simple to-synthesize siRNA carriers can rapidly adapt to target and silence oncogenes effectively in vivo. Besides enabling investigators to define a specific oncogenes’ role on tumor growth in vivo, this platform may be helpful for companies developing alternative therapeutic technologies. Targeting a specific gene with our siRNA delivery platform may facilitate deciding whether to proceed with small drug or antibody development. Thus, this HK delivery platform is complementary and does not necessarily compete with other tumor-targeting technologies. Without too much development, the current HK polyplexes could have a role in target validation for investigators and companies.

Therapeutics is the second area where these targeted HK siRNA polyplexes may have a role, particularly for NRP-1-expressing solid tumors with limited therapeutic options. Despite the likely short half-life of the unmodified polyplex [29], the milestone of developing a simple in-design siRNA carrier that is effective in a tumor-bearing mouse model has been met. The relevance of a mouse model to humans remains uncertain, but NRP-1-mediated polyplexes may not have the same rules for efficacy as EPR-mediated polyplexes (e.g., prolonged half-life) [29]. Several studies will be required to advance these polyplexes to clinical trials, including dose–response with safety/toxicity profiles, biodistribution, bioactivity in normal tissues, and pharmacodynamics. Our immediate interest in the carrier is targeting Raf-1, a key mediator of tumor growth and angiogenesis [52]. Because of Raf1’s role in angiogenesis, tumors have a reduced ability to develop resistance to siRNA targeting Raf-1 of tumor vessels [53]. Notably, Raf-1 reduction likely will lead to decreased PD-L1 expression on tumor and tumor endothelial cell surfaces [54]. Suppression of PDL-1 in tumor cells and tumor angiogenic endothelial cells by these polyplexes will augment NK and CD8 T cell activity [55]. Importantly, the current delivery technology will enable investigators to test and provide their insights into targeting the most sensitive oncogene for a particular tumor to advance therapeutics.

## Figures and Tables

**Table 1:** HK carriers.

HK Polymers	Sequences	Number and Content of a.a. in Linear or Terminal Branches ^1^
H2K	K-H-K-H-H-K-H-H-K-H-H-K-H-H-K-H-H-K-H-K ^2^	20-mer (8K, 12H)
H2K-CO_2_H	K-H-K-H-H-K-H-H-K-H-H-K-H-H-K-H-H-K-H-H-K-CO_2_H	21-mer (8K, 13H)
H3K-33	K-H-K-H-H-K-H-H-K-H-H-H-K-H-H-H-K-H-H-H- K-H-H-H-K-H-H-K-H-H-K-H-K	33-mer (11K, 22H)
H2K-P ^3^	[C-K-H-K-H-H-K-H-H-K-H-H-K-H-H-K-H-H-K-H-K-C]_X_	N.A. (8K, 12H, 2C)
H3K(+H)4b ^4^	[K-H-H-H-K-H-H-H-K-H-H-H-H-K-H-H-H-K]_4_LYS	18-mer (5K, 13H)
H2K4b-14	[K-H-H-K-H-H-K-H-H-K-H-H-H-K]_4_LYS	14-mer (5K, 9H)

^1^ Abbreviations, a.a.—amino acid; N.A.—not applicable. ^2^ Except for H2K-CO_2_H, other linear peptides and the branched LYS core have C-amide protection. ^3^ H2K-P formed by oxidizing C-H2K-C. The average number of concatemers (X) is about 6.6 [22]. ^4^ The four terminal branches of the HK polymers (H3K(+H)4b and H2K4b-14 emanate from a 3-lysine core.

**Table 2:** Luciferase inhibition of MDA-MB-435-Luc xenografts.

Polyplexes *	Inhibition
H2K-P (*n* = 8)	49.6 ± 12.8
H3K(+H)4b (*n* = 4)	13.8 ± 8.5
H2K (*n* = 3)	16.4 ± 9.4
H2K-PS ** (*n* = 8)	69.3 ± 5.8
H3K-33 ** (*n* = 3)	25.7 ± 7.3
H3K-33 (85%)/H2K-CO_2_H ** (*n* = 3)	78.8 ± 7.1
H3K-33 (75%)/H2K-CO_2_H ** (*n* = 3)	81.6 ± 5.0
H2K4b-14 ** (*n* = 3)	54.0 ± 15.9

* All HK/siRNA had a ratio of 1.5:1 (60 μg/40 μg, *w*:*w*). The ionizable N to P (N/P) ratios were as follows: H2K-P (2.1:1), H3K(+H)4b (2.0:1), H2K (2.3:1), H3K-33 (2.3:1) H3K-33 (85%)/H2K-C0_2_H (2.3:1), H3K-33(75%)/H2K-C0_2_H (2.2:1), H2K4b-14 (2.1:1). ** Sonicated HK polyplexes.

**Table 3:** Raf-1 PCR primer sequences.

Primer	Length	Sequence (5′-3′)
Raf-1-F	20	GTCCCAGCACTACCTTCTTT
Raf-1-R	22	AAGGCGTGAGGTGTAGAATATC

## Data Availability

The data presented in this study are available upon request from the corresponding author.

## Supplementary Materials

The following supporting information can be downloaded at: https://www.mdpi.com/article/10.3390/molecules29235541/s1. Figure S1, Gel Retention of H2K, H2K-P, and H3K(+H)4b siRNA Polyplexes—In Vitro. Figure S2, Gel Retardation for HK Polyplexes—In Vivo. Figure S3, Luciferase reduction in MDA-MB-231 xenografts with HK siLuc Polyplexes. Table S1, Biophysical Characteristics of HK Polyplexes—In Vivo.
