# Supplementary material for: A Facile and Promising Delivery Platform for siRNA to Solid Tumors
Source: Molecules. 2024 Nov 23;29(23):5541. doi: 10.3390/molecules29235541 (PMC11643702; doi:10.3390/molecules29235541)
Supplement: Supplementary file 1 [file molecules-29-05541-s001.zip › molecules-3237919-supplementary.pdf]

Supplementary Data

A Facile and Promising Delivery Platform for siRNA to solid tumors

Qixin Leng, Aishwarya Anand, and A. James Mixson

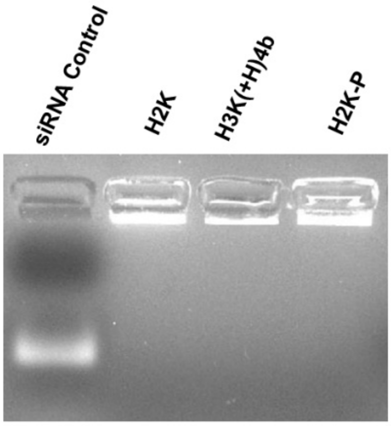

Figure S1. Gel Retention of HK siRNA Polyplexes-*In Vitro*. After the HK siRNA polyplexes (4 µg of HK: 1 µg of siLuc in 45 µl of Opti-MEM) were prepared in Opti-MEM for *in vitro* studies, their gel retentions were investigated. A sample of the polyplexes (25 µl, ~0.5 µg, of siRNA) was loaded onto a 3% agarose gel containing ethidium bromide (3.5 µl, 10 mg/ml), and electrophoresis was carried out in TAE buffer for 30 min at 50 mV.

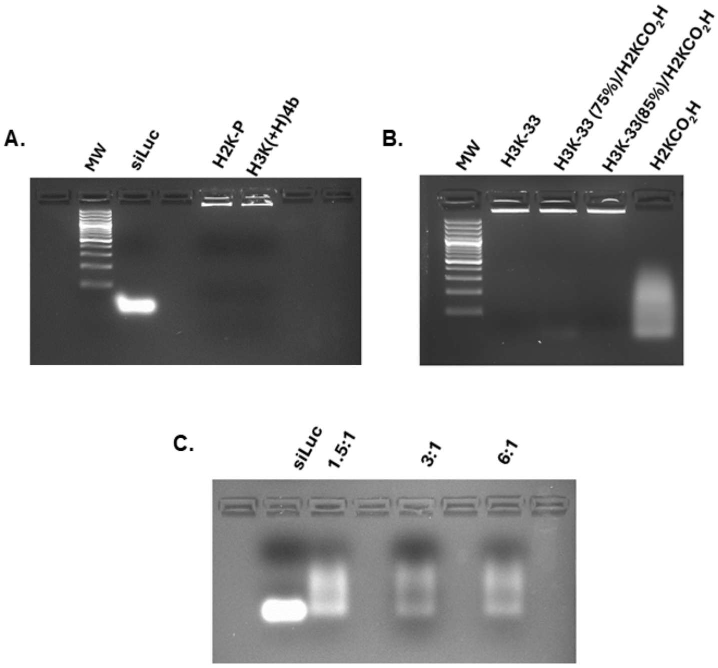

Figure S2. Gel Retention of HK polyplexes-*In Vivo*. **A, B.** After HK siRNA polyplexes were prepared as *in vivo* studies, their gel retention was investigated. The HK siRNA polyplexes were formed by mixing 60 µg of the peptide with 40 µg of siRNA in water (ratio, HK:siRNA, w: w, 1.5: 1). **C.** H2K siRNA polyplexes were prepared by mixing 240 µg, 120 µg, and 60 µg of the peptide with 40 µg of siRNA (ratio, HK:siRNA, w: w, 1.5:1, 3:1, 6:1). Forty minutes after mixing, a sample of the polyplexes (10 µl, ~1.74 µg of siRNA) was loaded onto a 3% agarose gel containing ethidium bromide (3.5 µl, 10 mg/ml), and electrophoresis was carried out in TAE buffer for 30 min at 50 mV. MW, molecular weight standard; siLuc, Luciferase siRNA control.

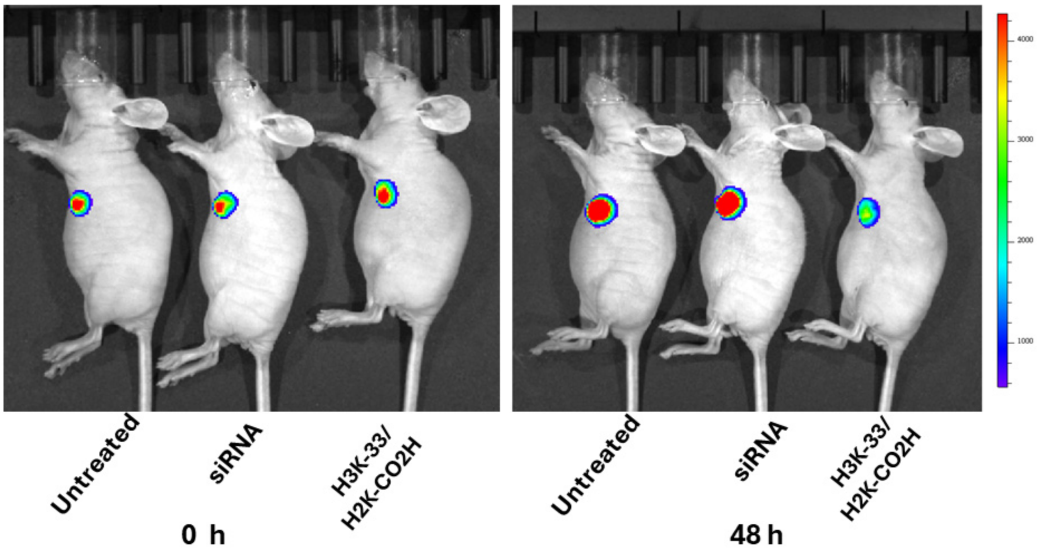

Figure S3. Luciferase reduction in MDA-MB-231 xenografts with HK siLuc Polyplexes. Like the siLuc polyplexes effects on MDA-MB-435-Luc, the H3K-33/H2K-CO_2H (15%) combination carrier of siRNA significantly reduced luciferase activity in the triple-negative MDA-MB-231-Luc tumors. While the untreated mice showed equivalent luciferase activity at the 0-time point, forty-eight hours later, there was a 71% reduction in luciferase expression with the combination carrier of siLuc compared to the siLuc control. The tumor sizes ranged from 113.8 to 114.6 mm^3 at 0 h and from 177.4 to 188.7 mm^3 at 48 h. Representative figure of two experiments.

Table S1. Biophysical Characteristics of HK Polyplexes-In Vivo.

**Polyplex**[1]	**Size (ZA)**[2]	**PDI**	**ZP (mV)**
H2K-P	106.9±5.2[3]	0.142±0.17	17.3±3.2
H3K(+H)4b	122.4±5.1	0.129±0.03	16.9±2.9
H2K	240.3±21.8	0.101±0.23	9.9±12.9
H2K-PS	112±17.7	0.132±0.12	11.3±3.1
H3K-33	291.8±11.3	0.133±0.18	14.6±5.9
H3K-33/H2KCO_2H (15%)	289.7±14.6	0.152±0.14	15.8±6.8
H3K-33/H2KCO_2H (25%)	325.4±18.4	0.121±0.11	8.4±4.5
H2K4b-14	168.7±7.5	0.157±0.15	5.9±5.7

1 Ratio of HK: siRNA (1.5:1; w/w)

2 Z-A is the Z-average size, PDI the polydispersity index, and ZP the zeta potential of the polyplex (see Materials and Methods."

3 Represent the mean ± SD
